# Extracellular vesicle-miRNAs as liquid biopsy biomarkers for disease identification and prognosis in metastatic colorectal cancer patients

**DOI:** 10.1038/s41598-020-60212-1

**Published:** 2020-03-04

**Authors:** Diego de Miguel Pérez, Alba Rodriguez Martínez, Alba Ortigosa Palomo, Mayte Delgado Ureña, Jose Luis Garcia Puche, Agustín Robles Remacho, José Exposito Hernandez, Jose Antonio Lorente Acosta, Francisco Gabriel Ortega Sánchez, Ma Jose Serrano

**Affiliations:** 1 GENYO, Centre for Genomics and Oncological Research, Pfizer/University of Granada/Andalusian Regional Government, Liquid biopsy and metastasis research group, PTS Granada, Avenida de la Ilustración 114, 18016 Granada, Spain; 2 Laboratory of Genetic Identification, Legal Medicine and Toxicology Department, Faculty of Medicine, University of Granada, Granada, Spain; 3 Integral Oncology Division, University Hospital Virgen de las Nieves, IBS Granada, Instituto de Investigación Biosanitaria de Granada, 18012 Granada, Spain; 4 Balearic Islands Health Research Institute (IdISBa), 07010 Palma de Mallorca, Spain; 5 Laboratory of Clinical Chemistry and Haematology, University Medical Center Utrecht, Utrecht, The Netherlands

**Keywords:** Tumour biomarkers, Tumour biomarkers, Colorectal cancer, Colorectal cancer

## Abstract

Disseminated disease is present in ≈50% of colorectal cancer patients upon diagnosis, being responsible for most of cancer deaths. Addition of biological drugs, as Bevacizumab, to chemotherapy, has increased progression free survival and overall survival of metastatic colorectal cancer (mCRC) patients. However, these benefits have been only reported in a small proportion of patients. To date, there are not biomarkers that could explain the heterogeneity of this disease and would help in treatment selection. Recent findings demonstrated that microRNAs (miRNAs) play an important role in cancer and they can be encapsulated with high stability into extracellular vesicles (EVs) that are released in biological fluids. EVs can act as cell-to-cell communicators, transferring genetic information, such as miRNAs. In this context, we aimed to investigate serum EV associated miRNAs (EV-miRNAs) as novel non-invasive biomarkers for the diagnosis and prognosis of Bevacizumab-treated mCRC patients. We observed that baseline miRNA-21 and 92a outperformed carcinoembryonic antigen levels in the diagnosis of our 44 mCRC patients, compared to 17 healthy volunteers. In addition, patients who died presented higher levels of miRNA-92a and 222 at 24 weeks. However, in the multivariate Cox analysis, higher levels of miRNA-222 at 24 weeks were associated with lower overall survival. Altogether, these data indicate that EV-miRNAs have a strong potential as liquid biopsy biomarkers for the identification and prognosis of mCRC.

## Introduction

Colorectal cancer (CRC) is the second most common cancer in women and the third most in men worldwide. Moreover, it accounts for 8.9% of all tumour-related mortality and is the second most common cause of cancer death^1^. Disseminated disease is present upon diagnosis in 50% of the patients [lymph nodes (35%) and distant organs (22%)] and half of the patients diagnosed as localized tumours will eventually develop it^2^. In recent years, the application of new targeted therapies, including anti-angiogenic drugs, has contributed to largely increase the overall survival (OS) of metastatic colorectal cancer mCRC patients, reporting median survivals of ≈30 months^3^. Bevacizumab, a humanized monoclonal antibody targeting vascular endothelial growth factor (VEGF) has demonstrated benefits in progression-free survival (PFS) and OS in combination with chemotherapy^4,5^. Despite these improvements in expectancy and quality of life and proven effectiveness of Bevacizumab, just 40% of patients exhibit favourable responses to the therapy^6^. Plasma/serum levels of VEGF-A^7^ and mutations in Ras/Raf/Mek/Erk pathway^8^, among others^9^, have been proposed as predictors of the efficacy of Bevacizumab. So far, controversial results have been reported^10–13^. Circulating tumour cells (CTCs), considered to be responsible for disease relapse, have been also evaluated as prognosis biomarker for mCRC patients under Bevacizumab therapy^14–16^, however more evidence is needed for clinical application.

This scenario creates an urgent need to discover and validate predictive biomarkers for prognosis and treatment response^17^. The use of liquid biopsy provides potential clinically-relevant non-invasive genomic and epigenomic signatures for cancer monitoring.

Due to their high abundance and their role as regulators of gene expression, circulating microRNAs (miRNAs), small non-coding RNAs (19–24 nucleotides long), have been proposed as potential markers in several cancer types^18^. Although, they are implicated in physiological processes, miRNAs take also part in cancer mechanisms including tumour growth, angiogenesis and metastasis. In cancer, miRNAs play two different roles that appear to be context specific, acting as onco-miRNAs when they inhibit the expression of tumour suppressor genes accelerating tumorigenesis or as tumour-suppressors when they prevent tumour progression by blocking oncogene expression^19^.

MiRNAs have been described in body fluids as circulating-free molecules^20^, associated with proteins^21^ or encapsulated in extracellular vesicles (EVs)^22^. Whereas circulating-free miRNAs isolated from body fluids include miRNAs released during cell death and damage^23^, miRNAs packaged in EVs are selectively released by cells for communication purposes. In addition, hypoxic conditions, that take place inside the tumour, enhance EVs release and modulate their content^24^. Thus, they might increase the amount of specific tumour miRNAs found in the blood^25^. Moreover, lipid membrane coverage protects miRNA from RNases degradation. As a result, EV-derived miRNAs present higher specificity and stability than circulating miRNAs, making them better liquid biopsy biomarkers for cancer diagnosis and prognosis.

**Figure 1:**
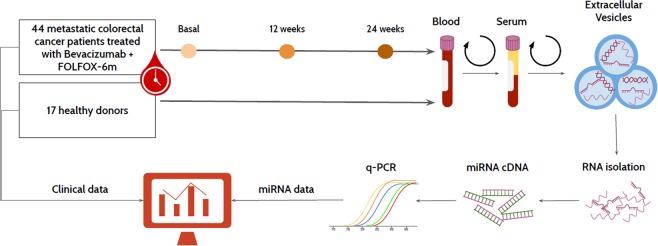
Graphical summary of the employed methodology during this study.

This study aimed to compare the potential of a selectively designed panel of EV-miRNAs with the commonly used clinical or experimental biomarkers as CEA, CTCs or KRAS status in the diagnosis and prognosis of mCRC patients under first-line Bevacizumab combined chemotherapy treatment (Fig. 1).

## Results

This study included 44 mCRC patients, 23 (52.3%) of them with primary tumours located in the colon and 21 (47.7%) in the rectum. Median follow-up time for all patients was 27 months (range 1 – 67 months). Clinical characteristics of patients and healthy donors are summarized in Table 1, including baseline CEA values that were also available in our cohort of healthy donors.

**Table 1:** Characteristics of metastatic colorectal cancer patients and healthy donors included in the study. Abbreviations: mCRC: metastatic colorectal cancer; 12w: 12 weeks; 24w: 24 weeks; CTCs: Circulating tumour cells.

Characteristics N (%)		mCRC patients (N = 44)	Healthy Controls (N = 17)		
**Gender**	Female	14 (31.8)	7 (41.2)		
	Male	30 (68.2)	10 (58.8)		
**Age (years)**	<55	13 (29.5)	15 (88.2)		
	> 55	31 (70.5)	2 (11.8)		
**Primary tumour location**	Colon	23 (52.3)			
	Rectum	21 (47.7)			
**Metastasis location**	Liver	23 (54.7)			
	Lung	13 (40)			
	Other	6 (14.3)			
**Metachronous metastasis**	Yes	5 (11.4)			
	No	39 (88.6)			
**Metastasis surgery**	Yes	11 (27.5)			
	No	29 (72.5)			
**K-RAS status**	Mutated	19 (46.3)			
	Wild-Type	22 (53.7)			
**CEA levels**		**Baseline**	**12w**	**24w**	**Baseline**
	Standard	10 (22.7)	15 (37.5)	15 (38.5)	16 (94.1%)
	High	34 (77.3)	25 (62.5)	24 (61.5)	1 (5.9%)
**CA 19.9 levels**		**Baseline**	**12w**	**24w**	
	Standard	19 (46.3)	22 (55)	22 (57.9)	
	High	22 (53.7)	18 (45)	16 (42.1)	
**Response**		**12w**	**24w**		
	Non-favourable	17 (43.6)	24 (60)		
	Favourable	22 (56.4)	16 (40)		
**Progression**	Yes	34 (79.1)			
	No	9 (20.9)			
**Death**	Yes	38 (86.4)			
	No	6 (13.6)			
**CTCs**	**Extraction**	**Baseline**	**12w**	**24w**	
	**Mean (range)**	**1.8 (0–17)**	**3 (0–64)**	**0.74 (0–6)**	
	Negative	27 (61.4)	24 (61.5)	27 (71.1))	
	Positive	17 (38.6)	15 (38.5)	11 (28.9)	

### Isolation and characterization of EVs

**Figure 2:**
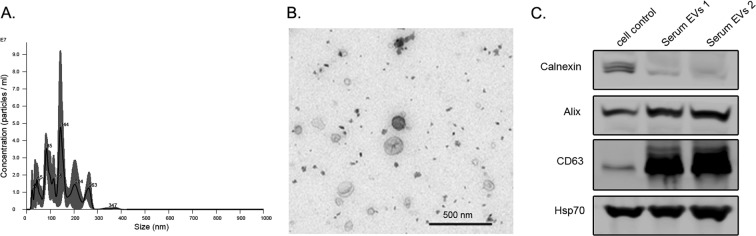
Characterization of EVs: (**A**) Nanoparticle Tracking Analysis analysis of extracellular vesicles from serum of healthy donors: Concentration 1.02×10^11^ ± 1.38×10^10^ and mode 127 nm ± 16 nm in size. (**B**) Transmission Electron Microscopy (TEM) image of isolated EVs from patients after purification via size exclusion chromatography (SEC), in order to reduce protein contamination in the staining. (**C**) Western blot image from cell control and serum EVs lysates, showing higher levels of calnexin, lower of Alix and lower of CD63 in cell compared to EV lysates. In addition, similar levels of Hsp70 could be observed between the 3 samples.

The study of serum EVs from cancer patients has become a very promising tool in the liquid biopsy field. Before performing specific analysis, the characterization of EVs is a mandatory step. According to the recommendations of the International Society of Extracellular Vesicles (ISEV), there are minimal requirements to claim a proper isolation of EVs^26^. In order to assess the population heterogeneity, the ISEV recommends that the employment of electron or atomic force microscopy be paired with a single tracking method. It also recommends the identification of specific markers as transmembrane proteins (CD63), intracellular proteins associated to membrane proteins (Alix, Hsp70), and intracellular proteins non-associated to plasmatic membrane proteins (Calnexin) by Western blot analysis. Following these requirements, we performed a precise characterization of our EVs samples. First, the Nanoparticle Tracking Analysis (Nanosight, Marvel. UK) resulted in a concentration of 1.02×10^11^ particles/ml and a diameter of mode = 127 nm ± 6.5 nm (Fig. 2A). Second, similar size and typical morphology of EVs were observed by transmission electron microscopy (TEM) (Fig. 2B). Lastly, the Western blot demonstrated high expression of Alix and CD63 on EVs samples, similar expression of Hsp70 on both culture cells and EVs, and higher levels of calnexin in culture cells compared to EVs (Fig. 2C). These data suggest that the employed methodology is suitable to isolate EVs from serum samples. Original blots are available in Supplementary Fig. S1.

### EV-miRNAs as biomarkers in mCRC identification

To evaluate the potential of the selected miRNAs to identify mCRC patients, we compared the baseline (B) serum expression of the 10 selected EVs-miRNAs in our cohort of 44 mCRC patients to the 17 healthy volunteers. The selection of these 10 miRNAs was based on previously described roles in the pathogenesis or having a CRC associated gene as a target. miRNAs analysed were miR-126, 155, 19b, 194, 20a, 200b, 21, 222, 552, and 92a (Supplementary Table S1).

**Figure 3:**
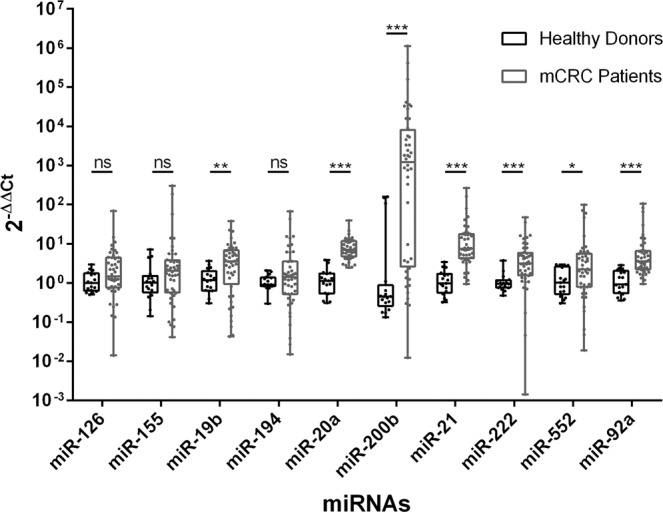
miRNA expression in mCRC patients and healthy donors: Extracellular vesicles (EVs) from metastatic colorectal cancer (mCRC) patients showed higher expression of miR-19b, 200b, 21, 222 and 92a in comparison to those of healthy donors. Data are presented as a box and whiskers plots (min to max). Mann-Whitney U test was used: *p < 0.05, **p < 0.01, ***p < 0.001, ns: no significant differences.

We identified statistically significant differences in 7 miRNAs, detecting higher levels of miR-19b (*p* = 0.008), miR-20a (*p* < 0.001), miR-200b (*p* < 0.001), miR-21 (*p* < 0.001), miR-222 (*p* < 0.001), miR-552 (*p* = 0.038) and miR-92a (*p* < 0.001) in mCRC patients (Mann-Whitney U test). However, miR-126 (*p* = 0.054), miR-194 (*p* = 0.343) and miR-155 (*p* = 0.131) showed no statistically significant differences between two groups (Fig. 3).

**Figure 4:**
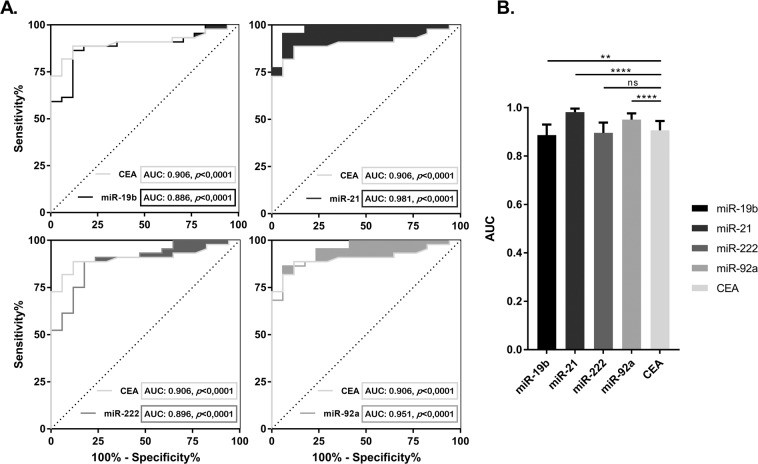
ROC curves and AUC of EV-miRNA expression in mCRC identification: (**A**) Receiver Operating Characteristics (ROC) curves and AUC of baseline EVs miR-19b, 21, 222, 92a and CEA, adjusted by age and gender, according to metastatic colorectal cancer (mCRC) identification. Shaded areas show the increased sensitivity and specificity of each miRNA vs. the CEA curve. (**B**) Histogram representing the AUC of each miRNA and their statistical differences with baseline CEA. miR-21 and miR-92 were the best predictors in the identification of mCRC with higher sensitivity and specificity than baseline CEA, while miR-222 showed no statistical differences and 19b had lower sensitivity and specificity than CEA. Data are presented as histogram of the mean with the standard error bars. *p < 0.05, **p < 0.01, ***p < 0.001, ****p < 0.0001, ns: no significant differences.

The univariate logistic binary regressions, adjusted by age and gender, showed that miR-19b (*p* = 0.029), miR-21 (*p* = 0.005), miR-222 (*p* = 0.026) and miR-92a (*p* = 0.005) were independent diagnostic factors of mCRC (Supplementary Table S2). We also evaluated their respective ROC curves to check the sensitivity and specificity in comparison with the current clinical biomarker CEA (Fig. 4A). The AUC of miR-21 and miR-92a were significantly higher than CEA AUC (*p* < 0.0001), while miR-222 and miR-19b presented a similar AUC (*p* = 0.147) and lower AUC (*p* = 0.0084) respectively (Fig. 4B) (Supplementary Table S2).

### EV-miRNAs vs clinical characteristics and CTCs

We compared the clinical characteristics and CTCs of our 44 mCRC patients with the expression of the 4 cancer-related EV-miRNAs (Table 2). The correlation with the other 6 EV-miRNAs has been presented in Supplementary table S3. We observed that high miR-222 (B) levels correlated with older patients (*p* = 0.017). K-RAS mutated patients showed low-levels of miR-92a (B) (*p* = 0.010) (Supplementary Figure S2). Furthermore, patients with elevated levels of CEA (B) expressed higher levels of miR-126 (*p* = 0.041) and miR-200b (*p* = 0.035) at baseline (data not shown). Low levels of miR-222 (B), were associated with high levels of Ca 19.9 at 24 weeks (24w), during second response assessment (*p* = 0.042), but no association was found between EV-miRNAs and other clinical variables. More interestingly, when we compared the expression of EVs-miRNAs and CTCs presence during the follow-up, we found a correlation between high levels of miR-21 (B) and the presence of CTCs (24w) (*p* = 0.023) (Table 2). Similarly, high levels of miR-92a at 12 weeks (12w), during first response assessment, were associated with presence of CTCs (12w) (*p* = 0.031) (data not shown).

**Table 2:** Association between baseline EV miRNA expression and mCRC patient characteristics. Mann-Whitney U and Kruskal-Wallis tests were used. Abbreviations: CTCs: Circulating tumour cells; miR: miRNA; B: Baseline; 12w: 12 weeks; 24w: 24weeks; *p < 0.05.

		**n**	miR-19b		miR-21		miR-222		miR-92a	
			median	*p*	median	*p*	median	*p*	median	*p*
**Gender**	Male	30	3.756	0.364	7.191	0.529	2.848	0.087	3.696	0.940
	Female	14	3.146		8.588		4.437		3.079	
**Age (years)**	<55	13	3.546	0.495	5.759	0.463	1.748	0.017*	2.886	0.389
	>55	31	4.455		7.831		4.762		3.616	
**Primary tumour location**	Colon	23	3.569	0.916	3.329	0.647	3.226	0.245	5.518	0.169
	Rectum	21	3.546		7.809		3.106		3.203	
**Metastasis location**	Liver	23	3.020	0.087	9.561	0.687	4.155	0.519	3.571	0.280
	Lung	13	4.986		7.155		2.661		2.439	
	Other	6	0.343		7.422		2.709		5.748	
**Metachronous metastasis**	No	39	3.820	0.386	7.809	0.216	3.478	0.802	3.571	0.858
	Yes	5	2.800		5.080		3.106		3.203	
**Metastasis surgery**	No	29	3.546	0.811	7.831	0.591	3.019	0.185	3.342	0.835
	Yes	11	3.569		5.759		5.101		3.185	
**K-RAS status**	Wild-type	22	2.789	0.053	7.724	0.347	4.768	0.143	3.696	0.010*
	Mutated	19	5.061		5.462		3.035		2.340	
**CEA (B)**	Standard	10	2.319	0.534	7.025	0.128	2.588	0.534	2.560	0.312
	High	34	3.882		13.151		3.352		3.696	
**CA 19.9 (B)**	Standard	19	3.271	0.855	7.227	0.448	3.117	0.734	2.681	0.182
	High	22	4.138		11.242		3.123		3.696	
**CEA (12w)**	Standard	15	3.943	0.699	6.822	0.376	4.155	0.192	2.681	0.720
	High	25	3.546		13.378		3.019		3.571	
**CA 19.9 (12w)**	Standard	22	3.420	0.527	7.242	0.840	3.817	0.240	3.194	0.840
	High	18	3.6883		10.377		2.481		3.524	
**CEA (24w)**	Standard	15	3.943	0.539	7.329	0.658	4.155	0.270	2.681	0.539
	High	24	3.557		13.151		3.027		3.673	
**CA 19.9 (24w)**	Standard	22	3.695	0.455	7.473	0.737	4.346	0.042*	3.035	0.781
	High	16	4.001		6.114		2.185		3.673	
**Response (12w)**	Non-favourable	17	3.569	0.747	7.227	0.547	3.035	0.510	3.775	0.362
	Favourable	22	3.146		7.219		3.817		2.667	
**Response (24w)**	Non-favourable	24	3.408	0.318	8.696	0.420	3.076	0.672	4.052	0.795
	Favourable	16	4.428		6.142		3.631		3.194	
**Progression**	No	9	4.455	0.353	6.822	0.268	5.681	0.471	2.681	0.736
	Yes	34	3.420		7.820		3.112		3.594	
**Death**	No	6	3.728	0.934	5.115	0.193	2.822	0.677	3.983	0.652
	Yes	38	3.557		7.713		3.171		3.524	
**CTC (B)**	Negative	27	3.943	0.392	12.923	0.477	4.155	0.604	3.616	0.539
	Positive	17	3.020		7.227		3.106		2.681	
**CTC (12w)**	Negative	24	3.557	0.943	7.473	0.875	4.246	0.146	2.9333	0.383
	Positive	15	4.455		6.822		2.301		3.571	
**CTC (24w)**	Negative	27	3.569	0.505	5.462	0.023*	3.019	0.251	2.439	0.308
	Positive	11	6.855		17.722		5.101		3.342	

### EV-miRNAs, CTCs, CEA and Ca19.9 in RECIST response prediction

RECIST responses (12w) were evaluated in 39 patients, 17 (43.6%) of them developed non-favourable responses while 22 (56.4%) patients exhibited favourable responses. RECIST response (24w) was evaluated in 40 patients, 24 (60%) patients presented non-favourable responses while 16 (40%) exhibited favourable responses (Table 1). We compared our clinical (CEA and Ca 19.9) and experimental biomarkers (EV-miRNAs and CTCs) with RECIST response during the follow-up to evaluate which was the best biomarker at predicting the response at 12 and 24 weeks. At baseline time, no clinical or experimental biomarkers were related to RECIST responses (*p* > 0.05). However, CEA (12w) was statistically correlated with the RECIST response (24w), resulting in higher CEA levels in those patients with non-favourable responses (*p* = 0.049).

### Progression-free survival

**Figure 5:**
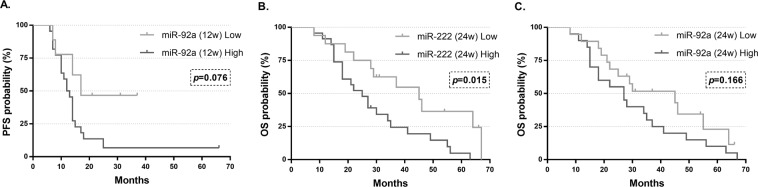
Kaplan-Meier curves of PFS and OS according to miR-92a and miR-222 expression: (**A**) Progression-free survival (PFS) probability curves according to lower and higher expression of miR-92 at 12 weeks (12w) showing increased DFS in the lower expression group but no statistical differences. (**B**) Overall survival (OS) probability curves according to lower and higher expression of miR-222 at 24 weeks (24w) are statistically different. (**C**) Overall survival (OS) probability curves according to lower and higher expression of miR-92 (24w) showing 2 separated curves but with non-statistically significant differences. Log-rank test was used. p = p-value.

Thirty-four of the 44 patients progressed during the study (77.3%). Median PFS of patients who progressed was 11 months (range: 1–63), compared to 34 (range: 7–66) months in those who did not progress. We evaluated the association between the clinicopathological characteristics, including our experimental biomarkers, and tumour-progression. This analysis showed that among all of these variables, only higher levels of miR-92a (12w) were associated with tumour-progression, although it was not statistically significant (*p* = 0.073) (Mann-Whitney U test). In the same way, when the miR values were dichotomized, the Kaplan-Meier analysis correlated higher risk of progression risk to patients with higher miR-92a (12w) levels (log-rank test *p* = 0.076) (Fig. 5A). In the univariate Cox’s regression analysis, we observed that the primary tumour location, the number of CTCs at baseline status and the expression of miR-92 (12w) were the only variables to be included in the model. However, in the multivariate analysis, none of them were an independent factor associated with PFS (*p* > 0.05) (Table 3). On the other hand, we analysed the role of these biomarkers as independent prognostic factors for PFS in wild-type (N = 22) and in mutated KRAS (N = 19) patients (Supplementary Table S4).

**Table 3:** Univariate and multivariate Cox proportional-hazards regression analysis for progression-free survival. Abbreviations: PFS: Progression-free survival; HR: Hazard Ratio; CI: Confidence Interval; p: p-value; CTCs: Circulating tumour cells; miR: miRNA; B: Baseline; 12w: 12 weeks; 24w: 24weeks; *p < 0.05.

			Univariate analysis			Multivariate analysis		
Characteristics		Median PFS (months)	HR	95% CI	*p*	HR	95% CI	*p*
**All patients**		11.5						
**Gender**	Female	11	1.17	0.55–2.47	0.688	1.62	0.60–4.33	0.339
	Male	14						
**Age (years)**	>55	13.5	0.75	0.36–1.56	0.436	0.49	0.16–1.46	0.198
	<55	10						
**Primary tumour location**	Colon	14	0.34	0.16–0.74	0.006*	0.47	0.18–1.25	0.131
	Rectum	10						
**Metastasis location**	Liver	13			0.509			
	Lung	12	0.94	0.43–2.09	0.885			
	Other	7	1.73	0.64–4.68	0.281			
**Metachronous metastasis**	Yes	12	1.67	0.63–4.42	0.301			
	No	11.5						
**Metastasis surgery**	Yes	14	0.82	0.36–1.85	0.630			
	No	11.5						
**K-RAS status**	Mutated	11.5	0.89	0.43–1.81	0.743			
	Wild-Type	13.5						
**CEA (B)**	High	11	1.33	0.58–3.06	0.509			
	Standard	13						
**Ca 19.9 (B)**	High	14	0.66	0.33–1.31	0.235			
	Standard	10						
**CEA (12w)**	High	11	1.46	0.69–3.09	0.324			
	Standard	14						
**Ca 19.9 (12w)**	High	13	0.66	0.31–1.38	0.267			
	Standard	13						
**CEA (24w)**	High	11	1.48	0.71–3.09	0.302			
	Standard	14						
**Ca 19.9 (24w)**	High	14	0.65	0.30–1.41	0.279			
	Standard	13						
**Response (12w)**	Favourable	12.5	0.61	0.30–1.25	0.181			
	Non-favourable	9.5						
**Response (24w)**	Favourable	14	0.67	0.33–1.36	0.263			
	Non-favourable	10						
**Number of CTCs (B)**			1.15	1.02–1.31	0.029*			
**Number of CTCs (12w)**			1.02	0.98–1.05	0.357			
**Number of CTCs (24w)**			0.98	0.75–1.28	0.896			
**miR-92a (12w)**	High	12	2.45	0.83–7.23	0.104	2.36	0.76–7.32	0.138
	Low	14						

### Overall survival

Thirty-eight patients died during the follow-up (86.4%), having a median OS of 23.5 months (range: 1–67), compared to 34 months (range: 28–66) of the 7 patients who survived. The association between the survival and the clinical variables, including our liquid biopsy biomarkers, was also analysed. Thus, the Mann-Whitney U test showed that higher levels of miR-222 (24w) and miR-92a (24w) were associated with the death of these patients (*p* = 0.009 & *p* = 0.024, respectively) (data not shown). As represented in Fig. 5B, when miR levels were dichotomized, there was a clear worse prognosis in patients with high levels of miR-222 (24w) (*p* = 0.015). On the contrary, despite visible differences in the curves, no statistical association was found between higher levels of this miRNA and a worse prognosis (*p* = 0.166) (Fig. 5C). In the Cox’s univariate analysis, miR-92a (24w) was not statistically associated with OS. However, high levels of miR-222 (24w), as well as the number of CTCs (12w), the clinical CEA levels (12w & 24w), and RECIST responses (12w & 24w) were all associated with worse prognosis (Table 4). The multivariate analysis presented the RECIST response and the miR-222 expression at 24 weeks as the only statistically associated variables with OS (*p* = 0.017 & *p* = 0.023, respectively) (Table 4). On the other hand, we analysed the role of these biomarkers as independent prognostic factors for OS in wild-type (N = 22) and in mutated KRAS (N = 19) patients (Supplementary Table S5).

**Table 4:** Univariate and multivariate Cox proportional-hazards regression analysis for overall survival. Abbreviations: OS: Overall survival; HR: Hazard Ratio; CI: Confidence Interval; p: p-value; CTCs: Circulating tumour cells; miR: miRNA; B: Baseline; 12w: 12 weeks; 24w: 24weeks; *p < 0.05.

			Univariate analysis			Multivariate analysis		
Characteristics		Median OS (months)	HR	95% CI	*p*	HR	95% CI	*p*
**All patients**		27						
**Gender**	Female	23.5	1.04	0.52–2.09	0.918	1.35	0.60–3.04	0.467
	Male	27						
**Age (years)**	>55	28	0.93	0.46–1.89	0.842	0.55	0.22–1.41	0.213
	<55	22						
**Primary tumour location**	Colon	27	0.95	0.49–1.84	0.883			
	Rectum	27.5						
**Metastasis location**	Liver	29			0.328			
	Lung	26	1.48	0.67–3.26	0.334			
	Other	18.5	2.07	0.74–5.78	0.167			
**Metachronous metastasis**	Yes	27	1.22	0.47–3.17	0.684			
	No	27.5						
**Metastasis surgery**	Yes	45	0.55	0.25–1.23	0.146			
	No	27						
**K-RAS status**	Mutated	30	0.73	0.36–1.47	0.374			
	Wild-Type	27						
**CEA (B)**	High	25	1.31	0.61–2.82	0.490			
	Standard	29						
**Ca 19.9 (B)**	High	26	1.07	0.54–2.11	0.848			
	Standard	29						
**CEA (12w)**	High	24.5	2.34	1.04–5.26	0.041*			
	Standard	37						
**Ca 19.9 (12w)**	High	24.5	1.58	0.76–3.29	0.222			
	Standard	35						
**CEA (24w)**	High	22	2.85	1.25–6.51	0.013*			
	Standard	37						
**Ca 19.9 (24w)**	High	26	1.62	0.75–3.45	0.220			
	Standard	35						
**Response (12w)**	Favourable	30.5	0.38	0.18–0.78	0.008*			
	Non-favourable	15						
**Response (24w)**	Favourable	36	0.356	0.17–0.76	0.007*	0.32	0.13–0.81	0.017*
	Non-favourable	18						
**Number of CTCs (B)**			1.12	0.98–1.28	0.089			
**Number of CTCs (12w)**			1.04	1.00–1.07	0.045*			
**Number of CTCs (24w)**			1.13	0.90–1.42	0.285			
**miR-222 (24w)**	High	25	2.55	1.16–5.58	0.02*	2.65	1.15–6.13	0.023*
	Low	34						
**miR-92 (24w)**	High	27	1.63	0.81–3.32	0.174			
	Low	30						

### Proteinase K and size exclusion chromatography controls

One of the major concerns of this work was the purity of EVs from serum samples. Serial centrifugation methods might also lead to the co-isolation of lipoproteins and proteins complex. More interestingly, these protein aggregates could be co-precipitated with serum circulating free-miRNAs. For this reason, to ensure that our results of miRNA expression were mostly related to EVs content, we performed two controls. The first control included a proteinase K treatment while the second consisted in the isolation of EVs by Size Exclusion Chromatography (SEC).

In the proteinase K control, neither miR-21 nor miR-222 showed statistical differences between treated vs. control paired samples in the Wilcoxon matched-pairs signed rank test (*p* = 0.25 & *p* = 1.00, respectively) (Supplementary Figure S3). Similarly, in the comparison between paired samples obtained by ultracentrifugation and by SEC, no statistical differences were found regarding miR-21 nor miR-222 expression (*p* = 0.5 & *p* = 0.25, respectively) (Supplementary Figure S4).

## Discussion

CRC is one of the most frequent causes of cancer death in the world^1^. In the last years, screening techniques have been improved, with special emphasis on blood based biomarkers^27^. Nevertheless, disseminated disease is still present at the time of diagnosis in a large percentage of patients, being responsible for most of these deaths. On the other hand, miRNAs have important roles on biological and pathological process in all the cell types. Particularly in cancer cells, specific miRNAs are actively encapsulated in EVs that are released to the circulation. In addition, due to the hypoxic microenvironment and/or alterations on endo-lysosomal trafficking, EVs production rates are relatively increased in tumour cells^28^. Considering the benefits of liquid biopsy as non-invasive with potential multiple sampling over time, especially important in the follow-up of metastatic patients, we thought that serum EV-derived miRNAs could serve as reliable and specific tumour biomarkers. The aim of the present study was to evaluate the disseminated disease identification and prognostic potential of our EV-miRNA panel in mCRC patients treated with anti-angiogenic therapy in combination with a FOLFOX regimen. This study included a homogeneous mCRC population treated with Bevacizumab-FOLFOX-6m, the standard first-line combination for those patients.

Our designed panel consisted of 10 miRNAs that had been previously described as markers associated with diagnosis (miR-155, 20a, 200b, 21, 222, 92a), prognosis (miR-155, 194, 20a, 21, 222, 552 and 92a), metastasis location (miR-19b, 194, 200b, 20a and 552), chemotherapy response (miR-126, 200b, 21), bevacizumab treatment (miR-126 and 155), and clinical stage (miR-21, 552 and 92a) (Supplementary Table 1). Our results showed the metastatic detection and prognostic value of each of these EV-miRNAs in our cohort of patients.

Based on our experiments, we were able to claim that our methodology successfully characterized miRNA expression of EVs cargo, with minimal contamination from circulating miRNAs that could be associated to EVs in protein aggregates.

EV-miRNA expression analysis from healthy subjects and mCRC patients showed miR-19b, miR-21, miR-222 and miR-92a as independent diagnostic factors with high specificity and sensitivity. The clear differences between the two groups demonstrate the potential use of these 4 miRNAs as biomarkers for early prediction of metastatic disease in CRC patients. In addition, miR-21 and 19b presented higher sensitivity and specificity than the routine clinical biomarker CEA.

Our results fall in line with previous studies on CRC where plasma or serum were employed as a sample for miRNA study. Chen *et al*. detected higher levels of serum miR-222 in CRC patients using Solexa sequencing^29^, and Giráldez, *et al*. found that miR-19b were significantly up-regulated in plasma from CRC patients compared to healthy donors^30^. As we observed in our results, the meta-analysis performed by Yang, *et al*. defined plasma miR-92a as a diagnostic marker for CRC^31^. Furthermore, Ogata-Kawata, *et al*. proved that serum exosome-derived miR-21 was an independent diagnostic factor^32^ and predictive factor for OS in all stages of CRC^33^. Our results reinforce those from Ostenfeld *et al*., that reported a significant higher expression of miR-222 in EpCAM^+^ EVs from CRC compared to healthy donors^34^, and from Fangfang *et al*. that correlated exosomal miR-92a expression to clinical stage of CRC patients^35^. Interestingly, despite including similar cohorts of patients, each of these studies identified a different miRNA as a diagnostic biomarker. These differences could suggest that the miRNA signalling depends on the disease status and/or the type of sample extracted (EV-miRNAs, EPCAM^+^ Exo miRNAs or free miRNAs).

The observed over-expression of miR-20a, miR-21, miR-222 and miR-92a are all in concordance with multiple evidence that demonstrate the importance of these miRNAs as tumour promoters. For example, it has been well documented that miRNA-21 targets a great number of key proteins: activated protein 1 (AP-1), nuclear factor I (NFI), maspin, fas ligand, programmed cell death protein 4 (PDCD4), tropomyosin 1(TPM1), tissue inhibitor of metalloproteinase 3 (TIMP3), acid nuclear phosphoprotein 32 A (ANP32A), phosphate and tensin homologue (PTEN), signal transducer and activator of transcription 3 (STAT3), c-Jun N-terminal kinase (JNK), extracellular signal-regulated kinase (ERK), mitogen associated protein kinase (MAPK),s and many others proteins with important roles in cancer^36^. However, due to the high homogeneity of our population and the high range of complex gene interactions of each miRNAs, we did not observe many significant associations between miRNA levels and clinical data. Other researchers investigated KRAS-dependence for miRNA selection as EVs cargo, where miR-10b was preferentially enriched in wild-type KRAS-derived exosomes, while miR-100 was enriched in mutant KRAS-derived exosomes^37^. Here, we observed lower levels of EVs-derived miR-92a in KRAS mutated compared with wild-type patients. This can be explained by the study conducted by Mackenzie *et al*.; their results showed that KRAS mutation reduces packaging of specific miRNAs into EVs through the reduction on Ago2 phosphorylation^38^.

Additionally, our data report an association between miRNA levels and the presence or absence of CTCs. During the last 20 years, CTCs have become an stronger marker for prognosis and response to the treatment^39,40^. CTCs are implicated in disease dissemination and they have been proposed as indicators of minimal residual disease^41^, which cannot be detected by standard imaging methodologies in the clinical practice^42,43^. miRNAs profiles have been related to the metastatic spread of the tumour, characterized by the presence of CTCs^44^. However, the methods for CTCs identification have not been completely standardized, including several expensive and time-consuming isolation and characterization protocols^45^. Our study, with a shorter turnaround time, proves that EV-miRNAs could become an important liquid biopsy tool to replace or complement the clinical relevance of CTCs.

Finally, in accordance with previous studies, our results report high expression of EV-miR-92a in patients with shorter PFS and OS, although not reaching enough statistical significance, probably due to the low number of patients. On the other hand, we observed that patients with higher EV-miR-222 levels presented shorter OS with clear statistical differences. Other studies have reported that the overexpression of miR-222 is related to poor survival in several types of cancer, including colorectal cancer^46^, as it has been associated to enhanced migration and invasion of cancer cells. In addition, our multivariate analysis presented miR-222 (24w) as a better prognostic factor for the overall survival than the clinical biomarkers CEA and Ca 19.9, as well as the CTCs that lost statistical significance during the analysis.

In conclusion, our data prove the importance of EV-miRNAs as potential liquid biopsy biomarkers in metastatic disease detection and the prognosis of overall survival of mCRC patients, complementing or even replacing the current clinical biomarkers CEA and Ca 19.9 and the CTCs.

### Limitations of the study

This study has two considerable limitations. First, our cohort included only 44 patients, what makes necessary to confirm these results in a larger case series. Second, we restricted our analysis to a specific panel of EV-miRNAs on the basis of literature and bioinformatics target results, however, we cannot exclude the possibility that other miRNAs may also have clinical relevance in our population.

## Methods

### Study design

This prospective longitudinal study included 44 mCRC patients who underwent first-line treatment with FOLFOX-6m (Oxaliplatin 85 mg/m2, Leucovorin 400 mg/m2, 5-fluorouracil (FU) 400 mg/m2 bolus and 5-FU 2400 mg/m2) over 46 h and Bevacizumab (5 mg/kg) every 2 weeks until disease progression, at the Department of Oncology, San Cecilio University Hospital in Granada (Spain), between April 2011 and November 2015. None of the patients had previously received any other type of biological treatment. A cohort of 17 blood donors with no history of malignant disease was recruited from the University of Balearic Islands, Mallorca (Spain). The study was conducted in accordance with the Declaration of Helsinki and approved by the ethical Committee of the Hospital. Written informed consent was obtained from every enrolled subject.

Computed tomography of the chest, abdomen and pelvis was performed at baseline, at 12 weeks (12w), at 24 weeks (24w) and finally each 12 weeks until death. Image interpretation was performed using Response Evaluation Criteria in Solid Tumours (RECIST) version 1.1^47^ to classify the patient evolution as complete response, partial response, stable disease, considered favourable responses, or as progressive disease, considered non-favourable response. Data were collected for the following clinical variables: age, gender, primary tumour location, metastasis surgery, primary tumour surgery, synchronous metastasis, K-RAS status, baseline, 12 weeks and 24 weeks Carcinoembryonic antigen (CEA) and Carbohydrate antigen 19.9 (Ca 19.9), RECIST response at 12 and 24 weeks, progression and survival. Clinical outcomes were evaluated in terms of PFS and OS. PFS was defined as the elapsed time from the start of the treatment to progression or death. OS was defined as the elapsed time from the start of the treatment to death.

### Blood samples and CTC isolation

Peripheral blood samples (10 ml in EDTA Vacutainer® tubes for CTCs and 5 ml in BD Vacutainer® SST™ II Advance tubes for serum) were extracted before the initiation of therapy (B) and subsequently at 12 weeks and 24 weeks after, coinciding with the RECIST response assessment. Five ml of peripheral blood were also collected from healthy donors. CTC enrichment and detection were performed according to protocols previously established by our group^48,49^. Briefly, samples were processed by density gradient centrifugation with Histopaque-1119 (Sigma-Aldrich, UK) at 700 ×*g* for 45 min to isolate the mononuclear cell fraction containing the CTCs. This fraction was incubated with the multi-cytokeratin-specific antibody microbeads (CK3–11D5) (that binds to clones 7, 8, 18 and 19) (Miltenyi Biotec, Germany) and the FITC-anti-cytokeratin antibody (CK3-6H5) (Miltenyi Biotec, Germany). Cells were passed through the MACS Cell Separation magnetic columns (Miltenyi Biotec, Germany) and the enriched cytokeratin positive cells were spun down onto polylysine-coated glass slides for subsequent fluorescent microscopy visualization and enumeration.

### EVs isolation

Blood samples were centrifuged at 1500 ×*g* for 15 min for serum collection. Then, serum was centrifuged at 10.000 ×*g* during 30 min to eliminate cellular debris. Supernatants were ultracentrifuged in 6 ml polyallomer ultracentrifuge tubes (Thermo Scientific, UK) in TFT 80.4 Rotor (Thermo Scientific, UK) at 100.000 ×*g* for 1 h at 4 °C. After that, supernatants were removed and EVs pellets were directly resuspended into the tube, either by adding RIPA lysis buffer, for Western blot protein controls, or homogenization buffer from the Maxwell 16 miRNA Tissue kit (Promega, USA), for subsequent miRNA analysis in patients. Samples were stored at −80 °C until further processing.

### Cell culture

MCF-7 cells were cultured in DMEM high glucose Glutamax (Gibco, Germany) supplemented with 10% of fetal bovine serum (Gibco, Germany), 100 U/ ml penicillin and 100 ng/ ml streptomycin (Gibco, Germany) in a humidified incubator with 5% CO2 at 37 °C.

### Nanoparticle tracking analysis

EV size distribution and concentration were measured using a NanoSight NS300 system equipped with an LM14 405 nm violet laser unit (Malvern Instruments, UK). EVs were diluted in PBS (1:500) for appropriate analysis and visualized at camera level 16 under control of a script, which included acquisition of 3 movies for 1 min at a fixed temperature of 22 °C. Analysis was performed using NTA 3.1 software. Detection threshold was set at 5 and other settings were kept at default.

### Transmission electron microscopy (TEM) analysis

First, EV suspensions were purified using SEC to eliminate protein background. Then, they were adsorbed on active-carbon coated grids for 10 min, washed and fixed for 15 min in a 2% paraformaldehyde and 0.2% glutaraldehyde solution. Grids were briefly rinsed with water and immediately transferred to drops of uranyl methyl cellulose pH 4.0 on a cooled metal plate for 5 min, picked up and dried at room temperature. Finally, grids were introduced in a FEI Tecnai™ F20 (ThermoFisher, USA) TEM for imaging.

### Western blot analysis

EV proteins were extracted adding 50 µl of RIPA lysis buffer (Sigma Aldrich, Germany) to the obtained EVs pellets or MCF-7 cells for cell controls. Lysates were centrifuged at 14.000 ×*g* and supernatants were collected and stored for protein determination. Protein concentration was quantified by BCA Protein Assay Kit (Thermo Scientific, UK), according with manufacturer’s instructions. Same amount of protein from each sample were loaded into precast SDS-PAGE gel (GE life science, UK) and run at 100 mV until optimal separation was obtained. Then, proteins were transferred to a PDVF membrane at 30 mV overnight. Plotted membranes were cut and incubated with primaries antibodies [anti-CD63 (clone MEM-259) (Abcam, UK), anti-Hsp70 (clone BB70) (ENZO Life Science, USA), anti-calnexin (clone AF18) and anti-Alix (clone 3A9) (Thermo Scientific, UK)] overnight at 4 °C and later with secondary IRDye 800CW anti-mouse antibody (LICOR, Germany) for 1 h at room temperature. Finally, membranes were revealed with an Odyssey infrared scanner.

### RNA extraction, reverse-transcription and qRT-PCR

EV-miRNAs were extracted and analysed according previous publications of our group^44^. Briefly, EVs miRNAs extraction was performed using the Maxwell® 16 miRNA Tissue Kit (Promega, USA). Complementary DNA was synthesized with the TaqMan™ Advanced miRNA cDNA Synthesis Kit (Applied Biosystems, UK) and miRNA expression levels were analysed in triplicate using TaqMan™ MicroRNA assay probes (Supplementary Table S1) and TaqMan™ Universal PCR Master Mix (Applied Biosystems, UK) according to manufacturer recommendations in an Applied Biosystems 7900HT Fast Real-Time PCR System (Applied Biosystems, UK).

### miRNA panel selection

We designed a panel of 10 miRNAs (126, 155, 19b, 194, 20a, 200b, 21, 222, 552, 92a) and miR-16 as the endogenous control. Normalization was performed according to the 2^-ΔΔCt^ method^50^. miRNAs were selected according to previously described association with migration, angiogenesis, metastasis, diagnosis and prognosis in CRC. They were also selected based on their reported mRNA targets of genes associated with bevacizumab, CRC and mCRC as VEGF-1, VEGF-2, KRAS, APC, TGFBR, PI3KCA and TP53^51^, using the starBase v3.0 database^52^ (Supplementary Table S1).

### Proteinase K treatment

First, three serum samples of 4 ml were divided in two aliquots of 2 ml each. They were centrifuged at 10.000 ×*g* during 30 min and the obtained supernatant was centrifuged at 100.000 ×*g* for 3 h. After that, the supernatant was discarded and the pellets were resuspended in 100 µl of digestion buffer (50 mM Tris-HCL; pH 8, 0,1 mM CaCl_2_, 3 mM DTT and 2.0 M Urea). Second, an aliquot of each sample was treated with proteinase K (Qiagen, Germany) to a final concentration of 0.25 mg/ml while the other was considered the untreated control. All aliquots were incubated at 37 °C during 30 min according for digestion, and at 70 °C during 15 min for Proteinase K inactivation. Finally, treated and untreated tubes were washed with PBS and centrifuged again at 100.000 ×*g* during 3 h. Exosome pellets were obtained and used for subsequent miRNA analysis.

### Size exclusion chromatography (SEC)

As in the proteinase K treatment, two aliquots of 2 ml from 3 serum samples were employed. All samples were centrifuged at 10.000 ×*g* during 30 min and then, supernatants were employed to EVs isolation; one aliquot from each sample by SEC and another by ultracentrifugation. In the SEC, 2 ml supernatant was injected into a SEC Hiprep™ 16/60 Sephacryl® S-400 HR column (GE Healthcare, USA) at a flow rate of 0.5 ml PBS/min as previously described^53^. Aliquots of 5 ml were collected and EVs-containing fractions were pulled down by 100.000 ×*g* centrifugation during 3 h. Aliquots for ultracentrifugation followed our previously described protocol. Exosome pellets were employed for miRNAs analysis.

### Statistical methods

Statistical analyses and graphs were performed using SPSS [SPSS Statistics for Windows, Version 22.0 (IBM Corp., USA)] and GraphPad Prism [Version 7.00 (GraphPad Software, USA)]. EV-miRNAs and CTCs were assessed as quantitative and dichotomous (miRNA: low/high and CTC: presence/absence) variables. miRNAs expression cut-offs were calculated with the Cutoff Finder web application^54^. The association between miRNAs and clinical characteristics or CTCs was evaluated using non-parametric Mann-Whitney U and Kruskal-Wallis tests. Paired samples controls were analysed by Wilcoxon matched-pair signed rank test. Logistic binary regression, receiver-operating characteristics (ROC) curves and the Area Under the Curve (AUC) were performed to test the sensibility and specificity of the miRNAs to identify mCRC patients. Categorical variables were compared by the Fisher’s exact test and correlations were measured by Spearman’s rank correlation. PFS and OS analyses were performed by log-rank test and by univariate and multivariate Cox Proportional-Hazards Regression. We applied the criterion of more than a 10% change in the coefficient estimate and *p* < 0.15^55^ for the selection of variables to be included in the multivariate model. *p* < 0.05 values were considered statistically significant.

## Supplementary information

Supplementary information

## Data Availability

The datasets generated during and/or analysed during the current study are available from the corresponding author on reasonable request.
