# Supplementary material for: Extracellular vesicle-miRNAs as liquid biopsy biomarkers for disease identification and prognosis in metastatic colorectal cancer patients
Source: Sci Rep. 2020 Mar 4;10:3974. doi: 10.1038/s41598-020-60212-1 (PMC7055306; doi:10.1038/s41598-020-60212-1)
Supplement: Supplementary file 1 — Supplementary information [file 41598_2020_60212_MOESM1_ESM.pdf]

Supplementary data

Extracellular vesicle-miRNAs as liquid biopsy biomarkers for disease identification and prognosis in metastatic colorectal cancer patients

Diego de Miguel-Pérez[1,2†], Alba Rodriguez-Martínez[1,2†], Alba Ortigosa-Palomo[1], Mayte Delgado[3], Jose L. Garcia-Puche[1,3], Agustín Robles-Remacho[1], José Exposito-Hernandez[3], Jose Antonio Lorente[1,2], Francisco Gabriel Ortega-Sánchez[4,5]* and Mª Jose Serrano[1,3]*

Supplementary Figure S1: *Original blots obtained during WB analysis. First well corresponds to control cells, while second and third to EVs samples from healthy donors. Blots from top to bottom are Calnexin, Alix, CD 63 and HSP70.*

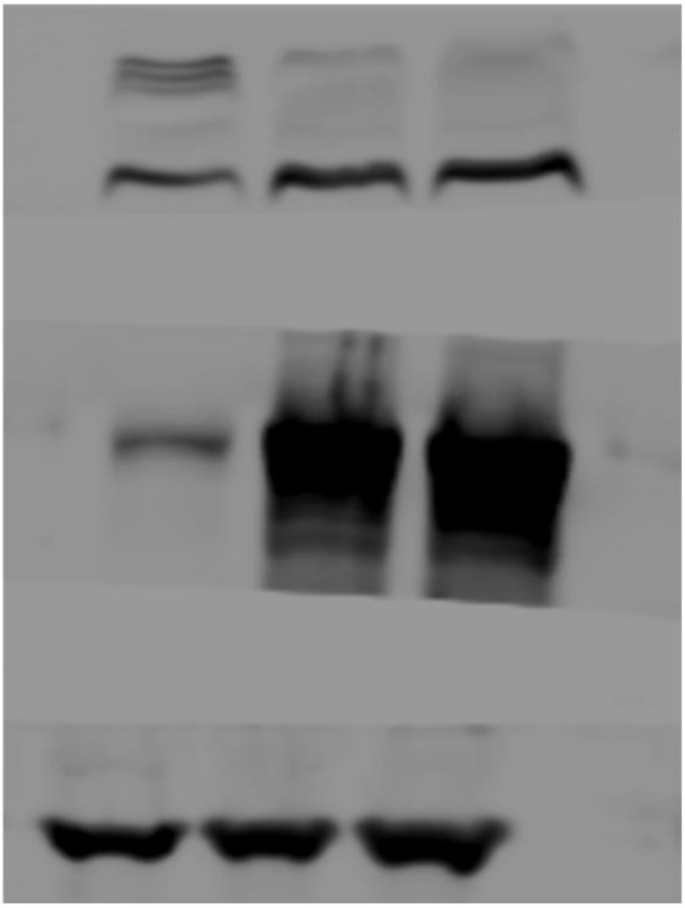

Supplementary Figure S2: *Baseline extracellular vesicles (EVs) miRNA expression according to K-RAS status in metastatic colorectal cancer (mCRC) patients. miR-92a showed statistically significant under expression in K-RAS mutated mCRC patients. Data are presented as a box and whiskers plots (min to max). Mann-Whitney U test was used:* $*p < 0.05$, $**p < 0.01$, $**p<0.001$, *ns: no significant differences.*

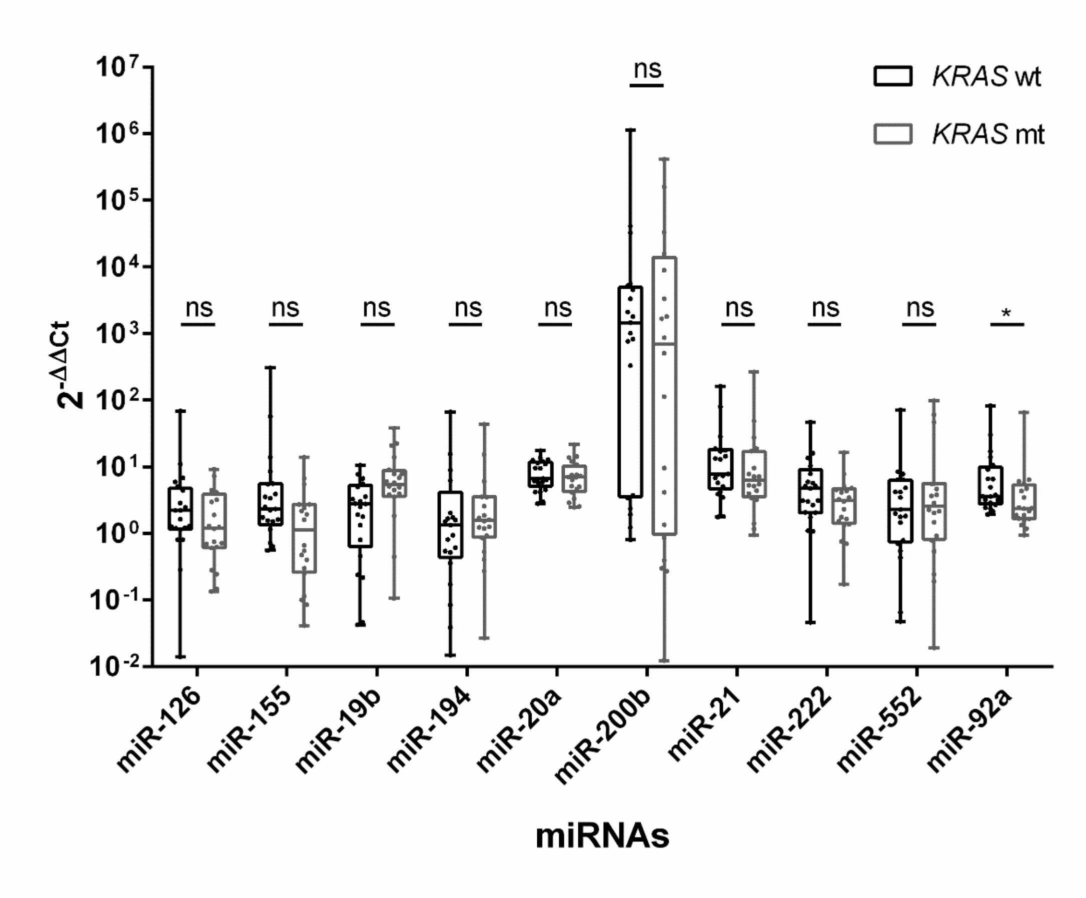

Supplementary Figure S3: *miRNA expression in control vs. proteinase K treatment. No significant differences were observed between paired samples extracted with ultracentrifugation vs. those extracted with ultracentrifugation plus proteinase K treatment. Data is presented as paired ΔCt of miR-21 and miR-222 vs miR16. Paired Wilcoxon test was used: ns: no significant differences.*

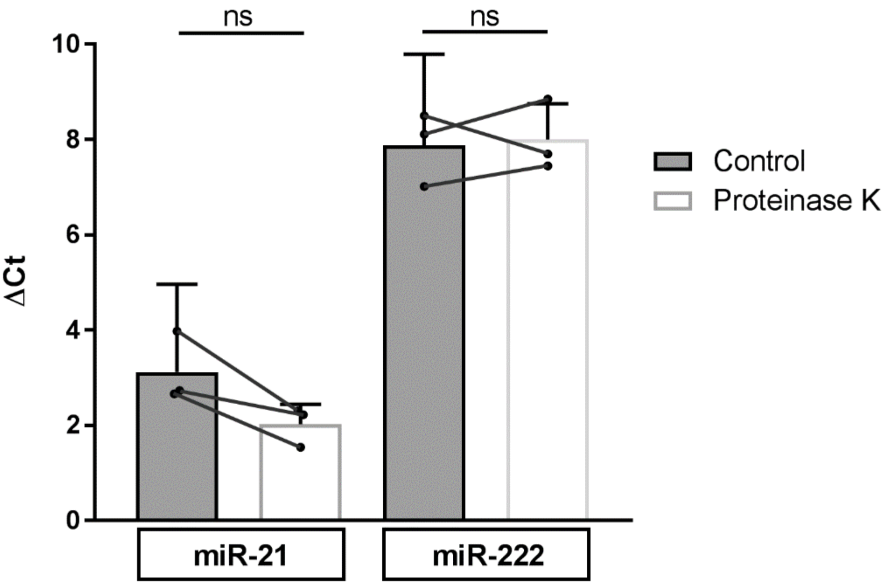

Supplementary Figure S4: *miRNA expression in ultracentrifugation vs. size-exclusion chromatography. No significant differences were observed between paired samples extracted with ultracentrifugation vs. size-exclusion chromatography. Data is presented as paired ΔCt of miR-21 and miR-222 vs miR16. Paired Wilcoxon test was used: ns: no significant differences.*

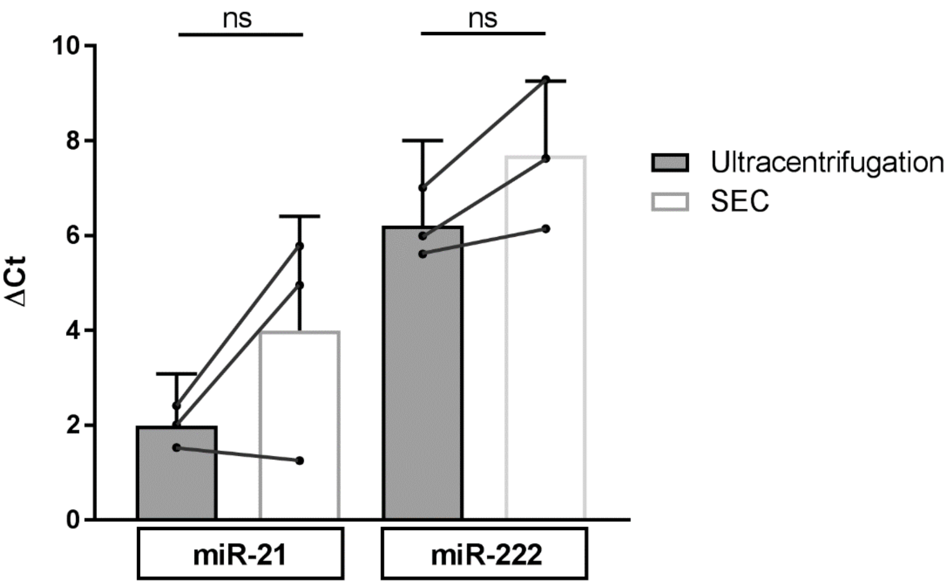

Supplementary Table S1*: Association of selected miRNAs and probe sequences.*

miRNA	Probe sequence	Cancer association	Reference
miR-126-5p	CAUUAUUACUUUUGGUACGCG	Bevacizumab and chemotherapy response	S1
miR-16-5p	UAGCAGCACGUAAAUAUUGGCG	Endogenous	S2, S3
miR-155-5p	UUAAUGCUAAUCGUGAUAGGGGU	Diagnosis, angiogenesis and prognosis in bevacizumab treatment	S4, S5, S6, S7
miR-19b-3p	UGUGCAAAUCCAUGCAAAACUGA	Liver and lung metastasis	S8
miR-194-5p	UGUAACAGCAACUCCAUGUGGA	Liver and lung metastasis and prognosis	S9
miR-20a-5p	UAAAGUGCUUAUAGUGCAGGUAG	Diagnosis, prognosis and metastasis	S4, S10, S11
miR-200b-3p	UAAUACUGCCUGGUAAUGAUGA	Diagnosis and liver metastasis, chemotherapy response	S12
miR-21-5p	UAGCUUAUCAGACUGAUGUUGA	Clinical staging, liver metastasis and prognosis	S4, S13, S14
miR-222-3p	AGCUACAUCUGGCUACUGGGU	Diagnosis, prognosis and metastasis	S15
miR-552-3p	AACAGGUGACUGGUUAGACAA	Clinical stage, lymph node metastasis and prognosis	S16
miR-92a-3p	UAUUGCACUUGUCCCGGCCUGU	Diagnosis, prognosis, clinical stage and metastasis	S4, S17, S18

Supplementary Table S2**:** *Logistic-binary regression and AUC of baseline EV miRNAs and CEA in mCRC identification. Analysis adjusted by gender and age of each miRNA revealed that only miR-19b, 21, 222 and 92a were independent identification factors of mCRC patients. In the comparison with baseline CEA, only miR-21 and miR-92a showed statistically higher AUCs (p<0.001). Abbreviations: HR: Hazard Ratio; CI: Confidence Interval; p: p-value; AUC: Area under the curve. EV: Extracellular vesicles. mCRC: metastatic colorectal cancer.*

	**Logistic-binary regression**			**ROC curves**			
**miRNA**	**HR**	**95% CI**	***p***	**AUC**	**95% CI**	***p***	**CEA comparison (*p*)**
miR-19b	1.86	1.07-3.24	0.029	0.886	0.80-0.97	<0.001	Lower (0.0084)
miR-20a	63.88	0.77-5292	0.065				
miR-200b	1.01	1.00-1.01	0.116				
miR-21	3.53	1.47-8.50	0.005	0.981	0.95-1.00	<0.001	Higher (<0.0001)
miR-222	2.56	1.10-4.61	0.026	0.896	0.81-0.98	<0.001	Similar (0.147)
miR-552	1.54	0.89-2.67	0.121				
miR-92a	8.56	1.89-38.69	0.005	0.951	0.90-1.00	<0.001	Higher (<0.0001)
CEA	1.53	0.98-2.40	0.062	0.906	0.83-0.98	<0.001	

Supplementary Table S3*: *Association between baseline EV miRNA expression and mCRC patient characteristics. Mann-Whitney U and Kruskal-Wallis tests were used. Abbreviations: CTCs: Circulating tumour cells; miR: miRNA; B: Baseline; 12w: 12 weeks; 24w: 24weeks; * p < 0.05.

		**n**	**miR-126**		**miR-155**		**miR-194**		**miR-20a**		**miR-200b**		**miR-552**	
			median	*p*	median	*p*	median	*p*	median	*p*	median	*p*	median	*p*
**Gender**	Male	30	1.284	0.614	1.671	0.465	1.292	0.364	6.370	0.096	1236.119	0.597	2.150	0.900
	Female	14	2.209		2.284		1.597		10.870		2076.639		2.809	
**Age (years)**	<55	13	1.215	0.495	1.153	0.169	1.511	0.807	6.382	0.563	331.136	0.108	1.463	0.263
	>55	31	2.205		2.335		1.181		7.483		1813.161		2.844	
**Primary tumor location**	Colon	23	1.901	0.805	2.233	0.518	1.608	0.217	7.549	0.916	3331.867	0.275	3.629	0.098
	Rectum	21	1.285		1.738		1.181		6.948		1010.280		1.474	
**Metastasis location**	Liver	23	1.901	0.874	2.143	0.619	1.588	**0.038***	7.248	0.434	2109.217	0.231	2.844	0.111
	Lung	13	1.644		2.408		1.608		6.359		1805.909		2.035	
	Other	6	1.062		1.088		0.272		5.808		384.021		0.663	
**Metachronous metastasis**	No	39	2.205	0.518	2.169	0.518	1.331	0.471	7.248	0.385	1676.611	0.448	2.271	0.134
	Yes	5	1.273		1.621		1.511		6.097		766.807		1.463	
**Metastasis surgery**	No	29	2.213	0.750	2.169	0.555	1.181	0.661	7.483	0.555	1805.909	0.209	2.160	0.916
	Yes	11	1.247		1.621		1.588		6.382		508.124		2.271	
***KRAS* status**	Wild-type	22	1.925	0.308	2.284	0.075	1.133	0.229	7.347	0.261	1236.119	0.676	2.865	0.754
	Mutated	19	1.247		1.604		1.608		6.948		864.989		2.265	
**CEA (B)**	Standard	10	0.857	**0.041***	1.378	0.355	1.597	0.575	6.523	0.737	167.423	**0.036***	1.176	0.170
	High	34	2.663		2.201		1.292		7.126		1961.189		1.557	
**CA 19.9 (B)**	Standard	19	1.284	0.834	1.930	0.695	1.522	0.979	6.551	0.063	331.136	0.060	1.474	0.060
	High	22	1.465		1.953		1.242		8.086		1741.260		3.699	
**CEA (12w)**	Standard	15	1.284	0.625	1.621	0.625	1.701	0.364	6.382	0.727	766.807	0.308	1.829	0.204
	High	25	2.865		2.233		1.181		7.483		1813.161		3.459	
**CA 19.9 (12w)**	Standard	22	1.773	0.664	2.188	0.480	1.517	0.703	6.805	0.724	637.466	0.211	1.652	0.219
	High	18	1.243		1.331		1.055		6.790		1569.284		2.947	
**CEA (24w)**	Standard	15	1.285	0.977	2.491	0.453	1.588	0.954	6.948	0.326	508.124	0.214	0.919	0.175
	High	24	2.052		1.940		1.217		6.682		2121.397		2.865	
**CA 19.9 (24w)**	Standard	22	2.053	0.636	2.320	0.183	1.517	0.906	7.126	0.165	637.466	0.214	3.151	0.836
	High	16	1.243		1.533		1.055		5.981		1741.260		2.212	
**Response (12w)**	Non-favourable	17	1.201	0.515	1.508	0.497	1.181	0.910	6.097	0.533	1461.958	0.755	2.265	0.713
	Favourable	22	1.593		2.049		1.555		7.366		795.096		2.439	
**Response (24w)**	Non-favourable	24	1.231	0.868	1.533	0.362	1.382	0.890	7.366	0.868	1569.284	0.868	2.865	0.320

	Favourable	16	1.773		2.049		1.460		6.606		1286.358		1.646	
**Progression**	No	9	2.205	0.511	2.491	0.811	1.608	0.531	6.551	0.221	5301.523	0.403	3.629	0.403
	Yes	34	1.465		2.036		1.242		7.126		1236.119		1.932	
**Death**	No	6	1.454	0.707	0.437	0.206	0.834	0.245	7.017	0.657	3083.256	0.945	2.832	0.682
	Yes	38	1.465		2.156		1.556		6.977		1236.119		2.212	
**CTC (B)**	Negative	27	2.461	0.555	1.930	0.933	1.252	0.952	6.662	0.588	2109.217	0.294	2.271	0.366
	Positive	17	1.273		2.143		1.522		7.248		823.386		1.829	
**CTC (12w)**	Negative	24	1.284	0.773	1.581	0.184	1.256	0.341	6.805	0.525	795.096	0.141	2.212	0.525
	Positive	15	1.644		2.335		1.608		7.248		1805.909		2.844	
**CTC (24w)**	Negative	27	1.273	0.253	1.621	0.064	1.331	0.359	6.382	0.430	864.989	0.961	2.265	0.784
	Positive	11	3.493		2.491		1.672		7.248		1676.611		2.271	

***Supplementary Table S4**: Univariate and multivariate Cox proportional-hazards regression analysis for progression-free survival. Data is represented only for variables that were included at least in one of the multivariate models (WT or mutated). Abbreviations: PFS: Progression-free Survival; HR: Hazard Ratio; CI: Confidence Interval; p: p-value; CTCs: Circulating tumour cells; miR: miRNA; B: Baseline; 12w: 12 weeks; 24w: 24weeks; * p < 0.05.*

		**K-RAS WT (N=22)**						**K-RAS mutated (N=19)**					
**PFS**		**Univariate analysis**			**Multivariate analysis**			**Univariate analysis**			**Multivariate analysis**		
**Characteristics**		**HR**	**95% CI**	***p***	**HR**	**95% CI**	***p***	**HR**	**95% CI**	***p***	**HR**	**95% CI**	***p***
**All patients**													
**Gender**	Female	0.74	0.24-2.28	0.598	2.27	0.34-15.19	0.397	2.26	0.64-8.03	0.206	3.22	0.70-14.90	0.135
	Male												
**Age (years)**	>55	1.48	0.42-5.27	0.541	0.24	0.03-2.08	0.194	0.57	0.18-1.80	0.337	0.118	0.02-0.65	**0.014***
	<55												
**Primary tumour location**	Colon	2.54	0.89-7.24	0.082	3.34	0.77-14.39	0.106	3.95	1.02-15.27	**0.047***			
	Rectum												
**Metastasis location**	Liver			0.226						**0.016***			**0.013***
	Lung	1.26	0.16-10.11	0.828				9.46	1.97-45.40	**0.005***	16.88	2.58-110.21	**0.003***
	Other	2.90	0.86-9.77	0.085				0.905	0.19-4.23	0.901	0.70	0.11-4.33	0.698
**Ca 19.9 (12w)**	High	0.92	0.33-2.54	0.867				0.374	0.11-1.25	0.110			
	Standard												
**CEA (24w)**	High	2.77	0.93-8.22	0.067				0.639	0.20-2.00	0.441			
	Standard												
**Ca 19.9 (24w)**	High	1.09	0.38-3.09	0.876				0.290	0.78-1.08	0.065			
	Standard												
**Response (12w)**	Favourable	0.44	0.15-1.32	0.143				1.26	0.27-5.87	0.769			
	Non-favourable												
**Response (24w)**	Favourable	0.39	0.13-1.14	0.085				1.33	0.44-3.99	0.609			
	Non-favourable												
**Number of CTCs (B)**		0.98	0.78-1.24	0.891				1.31	0.99-1.75	0.062	1.33	0.97-1.83	0.078
**miR-20a (B)**	High							5.47	1.35-22.09	**0.017***			
	Low												
**miR-20a (24w)**	High							4.01	1.02-15.68	**0.046***			
	Low												
**miR-222 (12w)**	High	0.24	0.06-0.91	**0.036***									
	Low												
**miR-19a (24w)**	High	0.23	0.07-0.75	**0.015***	0.14	0.03-0.74	**0.021***						
	Low												

Supplementary Table S5: *Univariate and multivariate Cox proportional-hazards regression analysis for overall survival. Data is represented only for variables that were included at least in one of the multivariate models (WT or mutated). Abbreviations: OS: Overall survival; HR: Hazard Ratio; CI: Confidence Interval; p: p-value; CTCs: Circulating tumour cells; miR: miRNA; B: Baseline; 12w: 12 weeks; 24w: 24weeks; * p < 0.05.*

		**K-RAS WT (N=22)**						**K-RAS mutated (N=19)**					
**OS**		**Univariate analysis**			**Multivariate analysis**			**Univariate analysis**			**Multivariate analysis**		
**Characteristics**		**HR**	**95% CI**	***p***	**HR**	**95% CI**	***p***	**HR**	**95% CI**	***p***	**HR**	**95% CI**	***p***
**All patients**													
**Gender**	Female	0.91	0.32-2.60	0.853	0.68	0.09-5.13	0.697	1.30	0.41-4.12	0.657	3.58	0.73-17.56	0.116
	Male												
**Age (years)**	>55	0.81	0.26-2.48	0.712	0.02	0.0-3.59	0.144	1.19	0.38-3.71	0.764	0.38	0.06-2.43	0.308
	<55												
**CEA (12w)**	High	2.58	0.91-7.31	0.074				1.84	0.47-7.18	0.378			
	Standard												
**CEA (24w)**	High	4.36	1.34-14.25	**0.015**				1.52	0.44-5.31	0.511			
	Standard												
**Response (12w)**	Favourable	0.57	0.21-1.51	0.258				0.29	0.07-1.24	0.094	0.08	0.01-0.94	**0.044***
	Non-favourable												
**Response (24w)**	Favourable	0.42	0.15-1.18	0.101				0.40	0.11-1.40	0.150			
	Non-favourable												
**Number of CTCs (12w)**		1.04	1.00-1.08	0.051	1.12	1.01-1.24	**0.032***	0.96	0.70-1.32	0.805			
**Number of CTCs (24w)**		0.90	0.61-1.35	0.622				1.35	1.00-1.81	**0.049***			
**miR-155 (B)**	High							10.45	1.94-56.27	**0.006***	19.39	1.18-319.50	**0.038***
	Low												
**miR-194 (B)**	High							3.85	1.19-12.44	**0.024***			
	Low												
**miR-552 (B)**	High							8.11	1.60-21.07	**0.011***			
	Low												
**miR-222 (24w)**	High							5.92	1.39-25.02	**0.016***	6.89	1.07-44.15	**0.042***
	Low												
**miR-19a (12w)**	High	5.92	1.37-25.56	**0.017***	17.15	1.01-291.59	**0.049***						
	Low												
**miR-194 (12w)**	High	6.32	1.04-38.34	**0.045***	607.87	2.03-182419.62	**0.028***						
	Low												

Supplementary references:

S1. Fiala, O. *et al.* The association of miR-126-3p, miR-126-5p and miR-664-3p expression profiles with outcomes of patients with metastatic colorectal cancer treated with bevacizumab. *Tumor Biol.* **39**, 1010428317709283 (2017).

S2. Wang, J. *et al.* Identification of a circulating microRNA signature for colorectal cancer detection. *PLoS One* **9**, e87451 (2014).

S3. Song, J. *et al.* Identification of suitable reference genes for qPCR analysis of serum microRNA in gastric cancer patients. *Dig. Dis. Sci.* **57**, 897–904 (2012).

S4. Hibner, G., Kimsa-Furdzik, M. & Francuz, T. Relevance of MicroRNAs as Potential Diagnostic and Prognostic Markers in Colorectal Cancer. *Int. J. Mol. Sci.* **19**, 2944 (2018).

S5. Lv, Z., Fan, Y., Chen, H. & Zhao, D. Investigation of microRNA-155 as a serum diagnostic and prognostic biomarker for colorectal cancer. *Tumour Biol.* **36**, 1619–25 (2015).

S6. Ulivi, P. *et al.* Circulating Plasma Levels of miR-20b, miR-29b and miR-155 as Predictors of Bevacizumab Efficacy in Patients with Metastatic Colorectal Cancer. *Int. J. Mol. Sci.* **19**, 307 (2018).

S7. Yang, D., Wang, J., Xiao, M., Zhou, T. & Shi, X. Role of Mir-155 in Controlling HIF-1α Level and Promoting Endothelial Cell Maturation. *Sci. Rep.* **6**, 35316 (2016).

S8. Pecqueux, M. *et al.* A Comprehensive MicroRNA Expression Profile of Liver and Lung Metastases of Colorectal Cancer with Their Corresponding Host Tissue and Its Prognostic Impact on Survival. *Int. J. Mol. Sci.* **17**, 1755 (2016).

S9. Chen, W.-Y. *et al.* The potential of plasma miRNAs for diagnosis and risk estimation of colorectal cancer. *Int. J. Clin. Exp. Pathol.* **8**, 7092–101 (2015).

S10. Cheng, D. *et al.* MicroRNA-20a-5p promotes colorectal cancer invasion and metastasis by downregulating Smad4. *Oncotarget* **7**, 45199–45213 (2016).

S11. Zhu, S.-H., He, X.-C. & Wang, L. Correlation analysis of miR-200b, miR-200c, and miR-141 with liver metastases in colorectal cancer patients. *Eur. Rev. Med. Pharmacol. Sci.* **21**, 2357–2363 (2017).

S12. Tsukamoto, M., Iinuma, H., Yagi, T., Matsuda, K. & Hashiguchi, Y. Circulating Exosomal MicroRNA-21 as a Biomarker in Each Tumor Stage of Colorectal Cancer. *Oncology* **92**, 360–370 (2017).

S13. Feng, Y.-H. & Tsao, C.-J. Emerging role of microRNA-21 in cancer. *Biomed. Reports* **5**, 395–402 (2016).

S14. Gao, H., Cong, X., Zhou, J. & Guan, M. MicroRNA-222 influences migration and invasion through MIA3 in colorectal cancer. *Cancer Cell Int.* **17**, 78 (2017).

S15. Xu, K. *et al.* MiR-222 modulates multidrug resistance in human colorectal carcinoma by down-regulating ADAM-17. *Exp. Cell Res.* **318**, 2168–77 (2012).

S16. Wang, N. & Liu, W. Increased expression of miR-552 acts as a potential predictor biomarker for poor prognosis of colorectal cancer. *Eur. Rev. Med. Pharmacol. Sci.* **22**, 412–416 (2018).

S17. Fu, F., Jiang, W., Zhou, L. & Chen, Z. Circulating Exosomal miR-17-5p and miR-92a-3p Predict Pathologic Stage and Grade of Colorectal Cancer. *Transl. Oncol.* **11**, 221–232 (2018).

S18. Chang, P.-Y. *et al.* MicroRNA-223 and microRNA-92a in stool and plasma samples act as complementary biomarkers to increase colorectal cancer detection. *Oncotarget* **7**, 10663–75 (2016).
